# Response to: Serum tissue polypetide-specific antigen (TPS): what is its diagnostic value – reply

**DOI:** 10.1054/bjoc.1999.1245

**Published:** 2000-04-27

**Authors:** F X Felberbauer, W Rebhandl

**Affiliations:** Department of General Surgery Department of Pediatric Surgery, General Hospital of Vienna, AKH-Wien, University of Vienna Medical School, Währinger Gürtel 18–20, Vienna, 1097, Austria


avoidance of radioactivity. We used both mostly as disease recurrence markers for adult patients with solid tumours, mainly breast and colorectal cancers, believing, as others, that this reflects the proliferative status of the particular tumour (Nekulova et al, 1995; Van Dalen et al, 1998; Nisman et al, 1998). We have abandoned the practice of monitoring patients with TPS (ASCO, 1997) after 7 months when we had completed 2703 tests. During this time we observed isolated elevation of TPS higher than 140 U $l^{-1}$, in 61 patients (breast and colorectal cancer), with other relevant markers (CA15-3, CEA, CA19-9) being below cutoff. In only four of them recurrence of disease was confirmed (breast cancer assessed by CA15-3, CEA, imaging techniques and complete examination by a medical oncologist). In two other patients (breast cancer) a suspect finding appeared on bone gallium scan, which was not subsequently confirmed on CT scan. Another two patients presenting with elevation of TPS were classified as stable disease (breast cancer, local partial remission). In another 55 patients restaging was performed at the discretion of a medical oncologist in charge, however, without contributing new information.

Especially data on TPS elevations approaching up to 2600 U $l^{-1}$ with no apparent disease progression in patients otherwise classified as ‘complete remission’ added significant stress to patients and their doctors and generated substantial unnecessary testing. Although inconclusive at this point, this group of false positives suffered mostly from chronic inflammatory and/or noninflammatory skin affections (herpetic infections, unhealed defects after radiotherapy with or without secondary bacterial infections and in some the reason was not apparent). In clinical practice the true difference between ‘false positivity’ and ‘lead time’ is difficult to distinguish during a limited time period; those markers with lead times longer than 7 months do not prove very useful for influencing patient outcome – this may obviously be the case of some of our 55 patients. It is our belief that the validity of new biomarkers and reevaluation of those used previously (Robertson, 1998) should be critically reassessed on a periodic basis if the ultimate goal is to improve patient care while avoiding unnecessary increases in noise and cost of health care. In agreement with the authors of the present paper, we currently recognize the potential of serum TPS only as a marker for monitoring response to cytotoxic therapy in explicitly defined diagnostic groups and when the potential of administering curative therapy exists, which indeed may include neuroblastoma and Wilms’ tumor. In our opinion, TPS should not be used as a diagnostic marker and only in exceptional cases as a marker for disease recurrence.

*D Valik, M Nekulova*
*Department of Biochemistry,*
*Masaryk Memorial Cancer Institute,*
*Zluty kopec 7, 656 53, Brno, The Czech Republic*
Supported in part by the Scientific and Research Scheme MZ00020980501.

#### REFERENCES

ASCO (1997) Update of recommendations for the use of tumor markers in breast and colorectal cancer. Adopted on November 7, 1997 by the American Society of Clinical Oncology. *J Clin Oncol* **16(2)**: 793–795

Caulin C, Salvesen GS and Oshima RG (1997) Caspase cleavage of keratin 18 and reorganization of intermediate filaments during epithelial cell apoptosis. *J Cell Biol* **138(6)**: 1379–1394

Einarsson R and Rylander L (1997) Tissue polypeptide-specific antigen (TPS) detects a specific epitope structure on human cytokeratin. *Anticancer Res* **17**: 3121–3124

Hannun YA (1997) Apoptosis and the dilemma of cancer chemotherapy. *Blood* **89(6)**: 1845–1853

Ishiwata I, Ono I, Ishiwata C, Soma M, Nakaguchi T, Ohara K, Hirano M and Ichikawa H (1991) Carcinoembryonic proteins produced by Wilms tumor cells in vitro and in vivo. *Experimental Pathology* **41**: 1–9

Janicke RU, Sprengart ML, Wati MR and Porter AG (1998) Caspase-3 is required for DNA fragmentation and morphological changes associated with apoptosis. *J Biol Chem* **273(16)**: 9357–9360

Mishaeli M, Klein B, Sadikov E, Bayer I, Koren R, Gal R, Rakowsky E, Levin I, Kfir B, Schachter J and Klein T (1998) Initial TPS serum level as an indicator of relapse and survival in colorectal cancer. *Anticancer Res* **18(3B)**: 2101–2105

Nekulova M, Pecen L, Eben K, Simickova M and Slama M (1995) Prediction of disease recurrence in oncological patients. *Neural Network World* **6(5)**: 945–950

Nisman B, Lafair J, Heching N, Lyass O, Baras M, Peretz T and Barak V (1998) Evaluation of tissue polypeptide specific antigen, CYFRA 21-1, and carcinoembryonic antigen in non-small cell lung carcinoma: does the combined use of cytokeratin markers give any additional information? *Cancer* **82(10)**: 1850–1859

Pronk LC, Stoter G, van Putten WL and de Wit R (1997) The correlation of CA15-3 and TPS with tumor course in patients with metastatic breast cancer. *J Cancer Res Clin Oncol* **123(2)**: 128–132

Rebhandl W, Rami B, Turnbull J, Felberbauer FX, Paya K, Bancher-Todesca D, Gherardini R, Mittlboeck M and Horcher E (1998) Diagnostic value of tissue polypeptide-specific antigen (TPS) in neuroblastoma and Wilms tumor. *Br J Cancer* **78(11)**: 1503–1506

Robertson JFR (1998) Blood tumour markers in breast cancer. *Tumour Marker Update* **10**: 31–37

Van Dalen A, Barak V, Cremaschi A, Gion M, Molina R, Namer M, Stieber P, Sturgeon C and Einarsson R (1998) The prognostic significance of increasing marker levels in metastatic breast cancer patients with clinically complete remission, partial remission or stable disease. *Int J Biol Markers* **13(1)**: 10–15

DOI: 10.1054/ bjoc.1999.1245, available online at http://www.idealibrary.com on IDEAL®

### Response to: Serum tissue polypetide-specific antigen (TPS): what is its diagnostic value – reply

**Sir**,

We are glad that our paper (Rebhandl et al, 1998) has aroused the interest of Drs Valik and Nekulova and that they can agree with our conclusions regarding the potential of TPS as a tool for monitoring therapy response. However, we would like to make a few comments on this letter.

First of all, our paper did not report clinical experience. The situation in paediatric oncology is very different from adult oncology. Apart from catecholamines and NSE in neuroblastoma (not in Wilms’ tumor) there are no ‘established’ tumour-markers. For TPS we actually had to establish normal values for healthy children (Rebhandl et al, 1997) before addressing patients with malignant disease. Furthermore, all our data are based on TPS and not on TPA, which in our opinion is not comparable.

Breast and colorectal cancer are among the most frequent malignant diseases in the western world, while the incidence of

© 2000 Cancer Research Campaign 

both neuroblastoma and Wilms' tumour is fortunately low. Large international multicentre studies are usually necessary to obtain sufficient data within an acceptable period.

For these reasons it is quite difficult to compare the applicability of TPS in so entirely different patient groups and different malignancies.

Any tumour marker (and any diagnostic tool) must be weighed against standard diagnostic procedures after exclusion of confounding factors. Again, it is too early to judge whether TPS measurements will become part of the diagnostic routine. In an otherwise healthy child presenting with an abdominal mass it could lead to an earlier diagnosis, especially since biopsy is strictly forbidden in (suspected) Wilms' tumours. ROC analysis enabled us to show good differentiation between benign and malignant disease in our patients.

The hypothesis that TPS could be a marker for apoptosis rather than for proliferation is interesting but it still awaits final experimental proof. TPS, which is a 22 kD fragment of cytokeratin 18 (Rydlander et al, 1996) may indeed correspond to the K18 C fragment described by Caulin (1997). Direct testing with the monoclonal antibody should decide this issue.

An activation of caspase-3 during chemo- or radiotherapy should actually lead to an *increase* in TPS-serum values and not to the significant decline we found in our patients. However, many issues are still undecided in this area, among them the surprising phenomenon that elevated TPS can be found in non-epithelial tumours (Vogl, 1995; Rebhandl, 1998. All this points to the fact that degradation of cytokeratin 18 may be a key element of tumour biology.

With our study we also wanted to encourage further studies of TPS and other tumour markers in paediatric malignancies.

*[1]FX Felberbauer[1] and W Rebhandl[2]*
*[2]Department of General Surgery,*
*Department of Pediatric Surgery, General Hospital of Vienna,*
*AKH-Wien, University of Vienna Medical School,*
*Währinger Gürtel 18–20, 1097 Vienna, Austria*

### REFERENCES

Caulin C, Salvesen GS and Oshima RG (1997) Caspase cleavage of keratin 18 and reorganization of intermediate filaments during epithelial cell apoptosis. *J Cell Biol* **138(6)**: 1379–1394

Rebhandl W, Felberbauer FX, Paya K, Rami B, Bancher-Todesca D, Bieglmayer C and Horcher E (1997) Tissue polypeptide-specific antigen in paediatric patients: assessment of normal values. *Med Ped Oncol* **29**: 218–221

Rebhandl W, Rami B, Turnbull J, Felberbauer FX, Paya K, Bancher-Todesca D, Gherardini R, Mittelboeck M and Horcher E (1998) Diagnostic value of tissue polypeptide-specific antigen (TPS) in neuroblastoma and Wilms' tumor. *Br J Cancer* **78(11)**: 1503–1506

Rebhandl W, Paya K, Felberbauer FX, Fuchs R, Henkel J, Bieglmayer C and Horcher E (1996) Tissue polypeptide-specific antigen (TPS) in paediatric malignancies. *Anticancer Res* **17**: 2865–2868

Rydlander L, Ziegler E, Bergman T, Schoberl E, Steiner G, Bergman AC, Zetterberg A, Marberger M, Bjorklund P, Skern T, Einarsson R and Jornvall H (1996) Molecular characterization of a tissue-polypeptide-specific-antigen epitope and its relationship to human cytokeratin 18. *Eur J Biochem* **241(2)**: 309–314.

Vogl M, Griesmacher A, Grimm M, Klepetko W and Muller MM (1995) Tissue polypeptide specific antigen for the detection of lymphoproliferative diseases induced by cyclosporin. *J Clin Pathol* **48(11)**: 1039–1044

 *© 2000 Cancer Research Campaign*

